# Pharmacokinetics of Piperacillin–Tazobactam in Critically Ill Patients with Open Abdomen and Vacuum-Assisted Wound Closure: Dosing Considerations Using Monte Carlo Simulation

**DOI:** 10.3390/pharmaceutics16091191

**Published:** 2024-09-09

**Authors:** Cédric Carrié, Jesse Butruille, Sophie Maingault, Alexandre Lannou, Vincent Dubuisson, Laurent Petit, Matthieu Biais, Dominique Breilh

**Affiliations:** 1 Surgical and Trauma Intensive Care Unit, Anesthesiology and Critical Care Department, Hôpital Pellegrin, CHU Bordeaux, 33076 Bordeaux, France; cedric.carrie@chu-bordeaux.fr (C.C.);; 2 Pharmacokinetics and Clinical Pharmacy Laboratory, University of Bordeaux, 33000 Bordeaux, France; 3 Clinical Pharmacy and City-Hospital Network Department, CHU Bordeaux, 33000 Bordeaux, France; 4 Digestive, Emergency and Trauma Surgery, CHU Bordeaux, 33000 Bordeaux, France; vincent.dubuisson@chu-bordeaux.fr

**Keywords:** piperacillin–tazobactam, population pharmacokinetics, open abdomen, Monte Carlo simulation, intensive care

## Abstract

Background: Open abdomen with vacuum-assisted wound closure therapy (OA/VAC) is frequently used in critically ill patients although the impact of OA/VAC on antibiotics pharmacokinetics (PK) remains unknown. We thus aimed to characterize the PK of piperacillin–tazobactam (PTZ) in critically ill patients with OA/VAC and assess the optimal dosing regimens based on pharmacodynamics (PD) target attainment. Methods: Over a 15-month study period, 45 patients with OA/VAC treated with PTZ administered continuously and adapted to 24 h creatinine clearance (CL_CR_) underwent measurements of free concentrations in their plasma, urine, VAC exudate, and peritoneal fluid. Population PK modeling was performed considering the effect of covariates, and Monte Carlo simulations were employed to determine the probability of target attainment (PTA) for the PK/PD targets (100% fT > 16 mg/L) in the plasma and at the peritoneal site at steady state. Results: Piperacillin concentrations were described using a two-compartment model, with age and total body weight as significant covariates for central volume of distribution (V1) and estimated renal function for clearance (CL). Tazobactam concentrations were described using a two-compartment model with estimated renal function as a significant covariate. The central volume of distributions V1 of piperacillin and tazobactam were 21.2 and 23.2 L, respectively. The VAC-induced peritoneal clearance was negligible compared to renal clearance. Most patients achieved the desirable PK/PD target when using a CL_CR_-pondered PTZ dosing regimen from 12 g/1.5 g/day to 20 g/2.5 g/day. Conclusions: Despite a wide inter-individual variability, the influence of OA/VAC on piperacillin and tazobactam PK parameters is not straightforward. The use of a CL_CR_-pondered PTZ dosing regimen from 12 g/1.5 g/day to 20 g/2.5 g/day is needed to reach a PTA > 85%.

## 1. Introduction

Early surgical source control and appropriate antimicrobial therapy (i.e., adequate dosing regimens and administration schemes) are cornerstones of the management of intra-abdominal infections (IAIs) [1]. According to current practice, piperacillin–tazobactam (PTZ) is one of the most frequently used antibiotics to treat IAIs in critically ill patients [2]. Piperacillin–tazobactam is a time-dependent antibiotic. Antimicrobial efficacy is correlated with the time the drug concentration in plasma remains above the MIC of the pathogen. This includes the free concentration in plasma and in tissues. For optimal efficacy, it is desirable for plasma and tissue concentrations be above the minimum inhibitory concentration (MIC) of the documented pathogen(s) for at least 100% of the time of the dosing interval (100% fT > MIC) [3]. On the other hand, pharmacokinetics and pharmacodynamics (PK/PD) can be altered in critically ill patients due to various pathophysiological changes, leading to important variability in the antibiotic concentrations and rates of therapeutic success [4,5].

**Figure 1:**
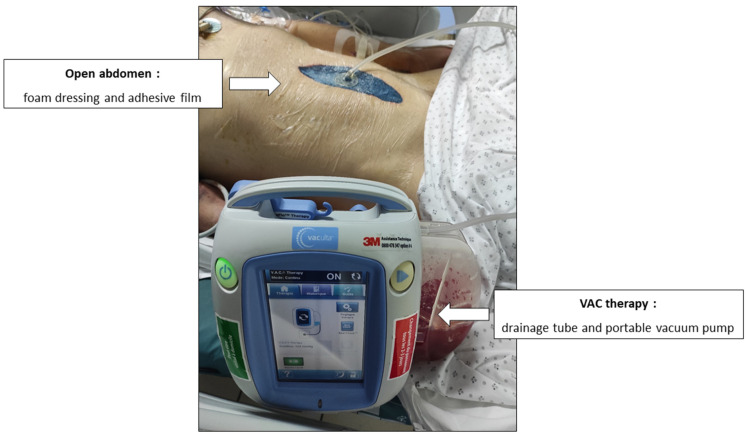
Description of open abdomen and vacuum-assisted wound closure.

For several years, open abdomen (OA) with temporary abdominal closure has been a surgical technique used primarily in emergency situations. It involves leaving the abdominal cavity open and temporarily covering it with a non-permanent closure to manage serious complications. The main indications for this approach are to treat or prevent intra-abdominal hypertension in critically ill surgical patients after abdominal compartment syndrome, major trauma, severe intra-abdominal infections, and other complications requiring ongoing management of the abdominal cavity [6]. In this context, vacuum-assisted wound closure therapy (VAC) is used to prevent contamination by draining abdominal fluids and clearing out inflammatory cytokines (Figure 1) [7]. Patients with an open abdomen are at high risk of serious complications that can lead to mortality due to the complexity of their clinical condition and the intensive care required. Causes of mortality mainly include severe infections, 20–50% [8]. Piperacillin–tazobactam is commonly used in medicine to treat serious infections, including those seen in intensive care patients or those requiring vacuum-assisted wound closure (OA/VAC) therapy. The use of OA/VAC therapy, which promotes the healing of complex wounds by negative pressure, may potentially influence the pharmacokinetics of antibiotics, including piperacillin–tazobactam. Specific studies on the effect of OA/VAC on the pharmacokinetics of piperacillin–tazobactam are limited. OA/VAC may improve the penetration of antibiotics into tissues surrounding the wound, thereby increasing their local efficacy. These are preliminary data that need to be consolidated through studies [9].

In this context, we hypothesized that OA/VAC should lead to significant changes in the volume of distribution (Vd) and/or drug clearance (CL) of piperacillin–tazobactam, potentially responsible for antibiotic underdosing. The main objective of this study was to characterize the population pharmacokinetics of piperacillin and tazobactam (PTZ) administered continuously in critically ill patients with OA/VAC and to assess the optimal dosing regimens for empirical MIC coverage by taking into account the covariates of interest (i.e., renal clearance) based on pharmacodynamics target attainment and Monte Carlo simulations.

## 2. Materials and Methods

### 2.1. Study Design, Protocol, and Population

This single-center, observational, and prospective study was conducted in a surgical and trauma Intensive Care Unit (ICU) at the Bordeaux University Hospital over a 15-month period (July 2021–November 2022). Every adult patient receiving an antimicrobial therapy with PTZ for a suspected or documented intra-abdominal infection while treated by OA/VAC during antimicrobial therapy were eligible for study enrollment. The exclusion criteria were a suspected length of stay ≤48 h and/or severe renal failure (CL_CR_ < 30 mL/min) without indication for continuous renal replacement therapy (CRRT). The study protocol was reviewed and approved by the Comité de Protection des Personnes Ile-de-France (IRB number: 2020-A03177-32). The patients and/or next of kin were informed about the inclusion in the current study, and none declined participation.

The dosing regimens and modalities of administration of PTZ were in accordance with a previously published protocol [10,11]. Briefly, PTZ was administered by continuous infusion (isotonic NaCl 0.9%, via a syringe, 50 mL, 12 h infusion, 2 infusions/day) after a loading dose of 4 g/0.5 g over 30 min. The dosing regimens were then adapted to the 24 h urinary measured creatinine clearance (CL_CR_): 16 g/2 g/day in patients with normal renal function and/or undergoing CRRT, 12 g/1.5 g/day in patients with moderate renal impairment (30 < CL_CR_ ≤ 60 mL/min), and 20 g/2.5 g/day in patients displaying augmented renal clearance (CL_CR_ > 150 mL/min).

Two sampling periods were planned in patients treated by OA/VAC after a first surgical source control:

T_0_: Serum sample (5 mL) collected 30 min after the loading dose of 4 g/0.5 g for therapeutic drug monitoring at peak concentration (C_max_). In patients already treated with PTZ before the first surgery, a serum sample was collected before administration of the loading dose to measure trough concentration (C_min_).

T_1_: Serum, VAC fluid, peritoneal fluid, and urinary samples (5 mL) were collected the day of the first scheduled surgical revision, within at least 24 h under OA/VAC and initiation of PTZ administered continuously. VAC fluid was collected through the drainage tube and the portable vacuum pump. The peritoneal exudate was directly collected in the operative room (OR) by the surgeon after removal of the adhesive film and the foam dressing.

All samples were centrifuged (3000 rpm for 15 min for removal of plasma and supernatant peritoneal fluid) and refrigerated at −80 °C until analysis. As previously described [4,12], an ultraviolet high-performance liquid chromatography (HPLC-UV) assay was used to measure the piperacillin (PIP) and tazobactam (TAZ) concentrations in plasma. Free (unbound) drug concentrations in the plasma and peritoneal fluid were determined for PIP and TAZ by ultrafiltration using a Millipore Microcon 30 kDa centrifugal filter unit (Merck KGaA, Darmstadt Germany). The mobile phase consisted of a linear gradient of ortho-phosphoric solution adjusted to pH 2 and acetonitrile from 3% to 55%, with a flow rate of 1 mL/min. Drugs were detected by HPLC-UV analysis using a C18 column (Hypersil™ C18, Fisher Scientific™, Illkirch, France) As recommended by the FDA Bioanalytical Method Validation guidelines, accuracy and reproducibility were evaluated for each monitored antibiotic, with coefficients of variation below 15% and accuracy between 85 and 115%. Linearity of the method was also checked with a coefficient of regression > 0.99 over the calibration range. The lower limit of quantification for unbound PIP and TAZ concentrations were, respectively, 0.5 µg/mL and 1.5 µg/mL [13].

### 2.2. Data Collection and Study Outcome

The unbound peritoneal concentration at steady state (Css_Pt_) was used to determine whether the optimal PK/PD target (100% fT _> MIC_) was achieved in critically ill patients with OA/VAC. An empirical target < 16 mg/L was used to define PIP underdosing, representing the highest MIC for Pseudomonas as per the European Committee on Antimicrobial Susceptibility Testing (EUCAST) [4]. For TAZ, empirical underexposure was defined by at least one sample under 4 mg/L, which represents the highest MIC for high-level β-lactamase producing strains [14,15,16,17,18,19,20,21,22,23,24,25]. We aimed to assess the adequacy of dosing in terms of the ‘worst-case scenario’, as type of pathogen and MIC data are often not initially available to the clinician prescribing an empirical (i.e., probabilistic) antimicrobial regimen.

Antibiotic infusion rate, urinary and VAC fluid volumes, and time between T_0_ and T_1_ were determined to measure urinary and VAC drug clearances, according to the following formula: CL_U_ or CL_VAC_ [L/h] = (amount of fluid or urine collected between T_0_ and T_1_ [L] x measured drug urinary or VAC concentration [mg/L])/(steady-state serum concentration [mg/L] x time between T_0_ and T_1_ [hours]). Peritoneal diffusion was calculated as the ratio of unbound peritoneal to plasma concentrations at steady state. Other covariates were collected at T_0_ and T_1_ and included the following: gender, age, total body weight (TBW), body mass index (BMI), simplified acute physiology score 2 (SAPS 2), reason for OA/VAC, type of IAI, modified sepsis-related organ failure assessment (SOFA) score (without neurologic and renal components), 24 h CL_CR_ or use of CRRT serum albumin, and amount of fluids collected daily by the VAC. For the 24 h CL_CR_, four groups were defined: group A for normal renal function (60 < CL_CR_ ≤ 150 mL/min), group B for patients who need CRRT, group C for moderate renal impairment (30 < CL_CR_ ≤ 60 mL/min), and group D for patients with augmented renal clearance (CL_CR_ > 150 mL/min).

### 2.3. Population Pharmacokinetic Modeling and Monte Carlo Simulations

Nonlinear mixed effect modeling of the concentration–time data using Monolix (version 2024R1, Lixoft, Antony, France) was performed. Since the goal is to study both tazobactam (TAZ) and piperacillin (PIP), we developed two pharmacokinetics (PK) models, sharing the same structural model (as the administration of both drugs always occurred in tandem) but with different statistical and error models. The outcome used to fit the PK models were the concentrations in plasma and peritoneal compartment. In both cases, the posology mainly consisted of a bolus followed by a continuous infusion (4 g/16 g of PIP and 0.5 g/2 g of TAZ in each dose) with an interval of 24 h hours between doses.

**Figure 2:**
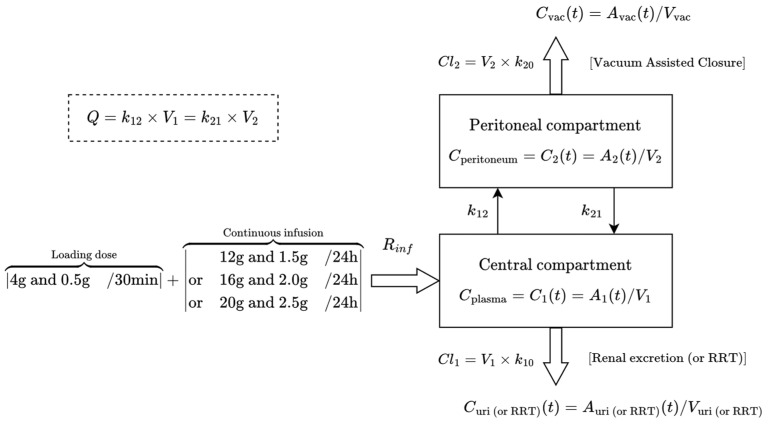
Theoretical two-compartment pharmacokinetic model for piperacillin and tazobactam. A_1_ and A_2_ (mg) = amount of drugs in the central and peritoneal compartments; V_1_ and V_2_ (L) = volume of distribution of the central and peritoneal compartments; *V_vac_* and *V*_*uri* (*or RRT*)_ = volume of VAC excretion fluid, urine, or renal replacement therapy (RRT); C (mg/L) = drug concentration in plasma, peritoneal fluid, VAC excretion fluid, and urine (or RRT); *Q*(h^−1^) = intercompartmental clearance; *k*_12_ and *k*_21_ (h^−1^) = transfer rate constants; *k*_10_ (h^−1^) = urine (or RRT) elimination rate constant; *k*_20_ (h^−1^) = VAC elimination rate constant on peritoneal fluid; *R*_*inf*_ (mg/h) = intravenous infusion rate.

The concentration–time data were analyzed using nonlinear mixed-effects modeling software program Monolix. The unbound serum and peritoneal drug concentrations were fitted to a theoretical two-compartment model with distribution factors to account for concentration differences between the plasma and peritoneal sites (Figure 2).

Monte Carlo simulations were used to evaluate the probability of target attainment (PTA) using various dosing regimens of piperacillin–tazobactam per day with the target of unbound piperacillin–tazobactam concentrations above the MIC for 100% of the dosing interval at steady state (100% fT > MIC). We evaluated target attainment stratified by renal function (groups A, B, and C).

The simulation data produced allow us to discuss the dosing regimens for piperacillin and tazobactam administered by continuous infusion with a loading dose at both the plasma and peritoneal fluid levels.

**Figure 3:**
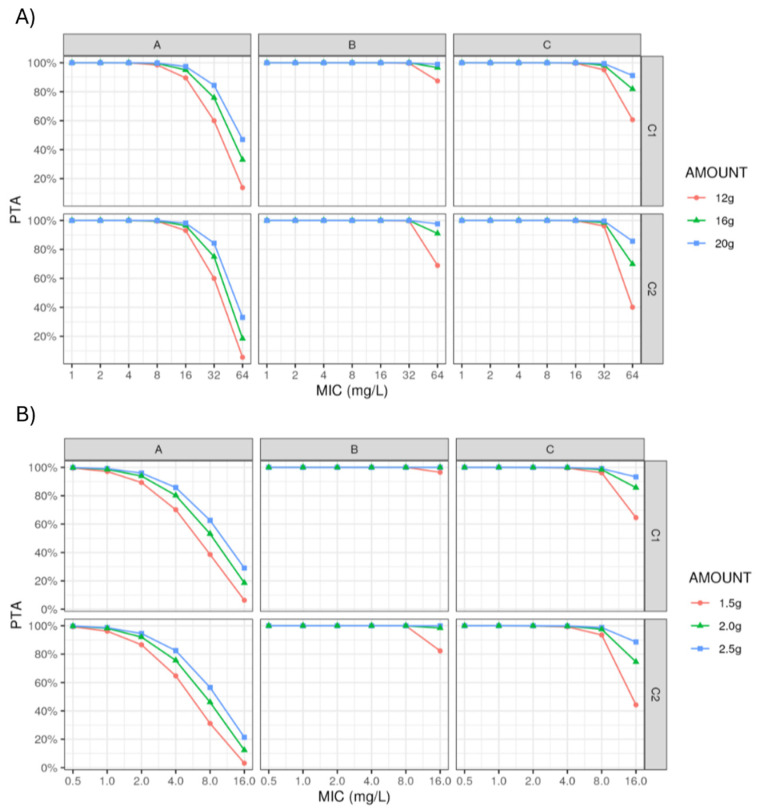
Probability of target attainment (100% fT>MIC) for piperacillin (**A**) and tazobactam (**B**) in central compartment concentration (C1) and peritoneal compartment concentration (C2) considering various renal function (normal renal function and augmented renal clearance [A], continuous renal replacement therapy [B], and moderate renal impairment [C]) and three dosing regimens (12 g/1.5 g/day, 16 g/2 g/day, and 20 g/2.5 g/day after a loading dose of 4 g/0.5 g).

Dosing simulations for piperacillin and tazobactam stratified by renal function (i.e., estimated creatinine clearance) are illustrated in Figure 3.

We discuss the results based on epidemiological cut off (ECOFF) values: 8 mg/L for Enterobacteriaceae and 16 mg/L for Pseudomonas aeruginosa and Enterococcus faecalis.

Population pharmacokinetic modeling and bootstrapping for PIP

A two-compartment model with linear elimination was retained, the proportional error model, with random effects on CL1 (elimination rate from the central compartment, here the plasma) and CL2 (elimination rate from the peritoneal compartment) and no assumed correlation. The age of the patients was used as a covariate on V1 and CL1, their weight as a covariate on V1, and the categorical clearance (creatinine clearance measured in mL/min) as a covariate on Cl (Table 1 and Equation (1)). Groups A and D were combined as a reference, given that they are closer in terms of values and also because an estimation of group D as a covariate failed due to the small size of the group (Table 2). V2 was fixed at 5 L with no random effects. Q (also with no random effects) and V1 were estimated with the maximum a posteriori (MAP), both values around 20 (L/h or L), with standard errors of 3 for Q and 1 for V1, both using the normal distribution as the maximum a priori. CL1 and CL2 were estimated with the maximum likelihood (MLE). All parameters follow a log-normal distribution (Equation (1)).

(1) $$\begin{array}{c} \log V_{1}=\log V_{1,pop}+\beta_{V_{1},age}\cdot {age}_{ind}+\beta_{V_{1},weight}\cdot {weight}_{ind}+\eta_{ind,V_{1}},\;\eta_{ind,V_{1}}∼N(0,\omega_{V_{1}}^{2})\\ \log V_{2}=\log V_{2,pop}\\ \log Q=\log Q_{pop} \end{array}$$

$$\begin{array}{c} \log Cl1=\log C{l1}_{pop}+\beta_{Cl_{1},age}\cdot {age}_{ind}+\beta_{Cl_{1},cl_{cat}=B}\cdot {cl_{cat\left[cat=B\right]}}_{ind}+\beta_{Cl_{1},cl_{cat}=C}\cdot {cl_{cat\left[cat=C\right]}}_{ind}+\eta_{ind,Cl1}\\ +\eta_{occ,Cl1},\;{\;\eta }_{ind,Cl1}∼N\left(0,\;\omega_{Cl1}^{2}\right),\;\eta_{occ,Cl1}∼N(0,\gamma_{Cl1}^{2})\\ \log Cl2=\log C{l2}_{pop}+\eta_{ind,Cl2},\;\eta_{ind,Cl2}∼N\left(0,\;\omega_{Cl2}^{2}\right) \end{array}$$

The residual error model chosen was the proportional model with a normal distribution [9,15]. The final parameter estimates underwent bootstrapping to derive the confidence intervals. We used 1000 repetitions with the Monolix R library Rsmlx (version 2024.1.5).

Population pharmacokinetic modeling and bootstrapping for TAZ

As for PIP, we retained a two-compartment model with linear elimination, the proportional error model, with random effects on CL1 (elimination rate from the central compartment) and CL2 (elimination rate from the peritoneal compartment) and no assumed correlation. The categorical clearance (creatinine clearance measured in mL/min) was used as a covariate on Cl (Table 3 and Equation (2)). Groups A and D were combined as a reference just like in the PIP model. V2 and Q were fixed to 5 and 20, respectively, with no random effects; V1 was estimated with maximum a posteriori (MAP) using the normal distribution centered around 20 with a standard deviation of 5 as the maximum a priori; and Cl1 and Cl2 were estimated with the maximum likelihood (MLE). All parameters follow a log-normal distribution (Equation (2)).

(2) $$\begin{array}{c} \log V_{1}=\log V_{1,pop}+\eta_{ind,V_{1}},\;\eta_{ind,V_{1}}∼N(0,\omega_{V_{1}}^{2})\\ \log V_{2}=\log V_{2,pop}\\ \log Q=\log Q_{pop}\\ \log Cl1=\log C{l1}_{pop}+\beta_{Cl_{1},cl_{cat}=B}\cdot {cl_{cat\left[cat=B\right]}}_{ind}+\beta_{Cl_{1},cl_{cat}=C}\cdot {cl_{cat\left[cat=C\right]}}_{ind}+\eta_{ind,Cl1}+\eta_{occ,Cl1},\;\eta_{ind,Cl1}\\ ∼N\left(0,\;\omega_{Cl1}^{2}\right),\;\eta_{occ,Cl1}∼N(0,\gamma_{Cl1}^{2})\\ \log Cl2=\log C{l2}_{pop}+\eta_{ind,Cl2},\;\eta_{ind,Cl2}∼N\left(0,\;\omega_{Cl2}^{2}\right) \end{array}$$

The residual error model chosen was the proportional model with a normal distribution [9,15]. The final parameter estimates underwent bootstrapping to derive confidence intervals. We used 1000 repetitions with the Monolix R library Rsmlx (version 2024.1.5).

Statistical comparison of the nested models was undertaken using log-likelihood ratios. If inclusion of the covariate resulted in a statistically significant improvement in the log-likelihood values (*p* < 0.05) and/or improved goodness-of-fit plots, then it was included. Goodness of fit was assessed by linear regression with an observed-predicted plot (individual and population), coefficients of determination (individual and population-weighted residuals [WR] and normalized prediction distribution error [NPDE] as a function of time and population prediction), and log-likelihood values. Using the final covariate model, a corrected visual predictive check (VPC) was performed (Supplementary Materials).

For each PTZ dosing regimen (12 g/1.5 g/day, 16 g/2 g/day, and 20 g/2.5 g/day), the duration for which the unbound drug concentration was above the minimum inhibitory concentration (fT > MIC) in the serum and peritoneal fluid was predicted. Probabilistic estimates of different PK/PD target attainments were assessed using Monte Carlo simulations (*n* = 10,000) for various MICs (1 to 64 mg/L for PIP and 0.5 to 16 for TAZ) and various renal functions (need for RRT, renal failure, normal renal function, and ARC). The groups normal renal function and ARC were combined as a reference given that they are closer in terms of values and also because an estimation of the ARC category as a covariate failed due to the small size of group A; a dosing regimen was considered successful if the fractional target attainment was  >85% [16].

*Monte Carlo simulations*

Monte Carlo simulation is a statistical technique used to model and analyze uncertain or complex systems. It relies on generating many random realizations of a model to estimate the properties of a phenomenon or system. The objectives of a Monte Carlo simulation are as follows:

Estimation of Risks and Uncertainties: One of the main uses of Monte Carlo is to assess the impact of uncertainties and random variations on the results of a model.

Prediction of Complex Behaviors: A Monte Carlo simulation is useful for predicting the behavior of complex systems that cannot be easily analyzed analytically due to its complexity or the large number of variables involved.

Optimization of Decisions: By simulating different scenarios and analyzing their results, decision makers can choose optimal strategies while taking into account variability.

Sensitivity Analysis: It helps identify which variables or factors influence the results the most.

Model Validation: By comparing the simulation results with real data or other models, the validity and accuracy of the models used can be determined.

Optimizing antibiotic doses in intensive care patients is a major challenge, given the pathophysiological variations that can affect drug pharmacokinetics (PK) and pharmacodynamics (PD). A Monte Carlo simulation is a powerful tool to optimize these doses in a personalized manner, taking into account individual variations [23].

Using the final pharmacokinetic models, we ran 10,000 Monte Carlo (MC) iterations for each substance (PIP and TAZ) and each clearance category (A, B, C, and D, the latter merged into group A, as explained above). We used the Simulx software of the Monolix suite (version 2024R, Lixoft, Antony, France). We were interested in calculating the probability of target attainment (PTA), which is the percentage of Monte Carlo iterations where the concentration was above the minimum inhibitory concentration (MIC) at steady state. The simulated treatments consisted of 30 min bolus doses (4 g PIP/0.5 g TAZ) and three 24 h infusion doses (12 g, 16 g, and 20 g PIP/1.5 g, 2 g, and 2.5 g TAZ). The MIC values we used to compute the PTAs were 0.125, 0.25, 0.5, 1, 2, 4, 8, and 16 mg/L for TAZ and 1, 2, 4, 8, 16, 32, and 64 mg/L for PIP. The fractional target attainment (FTA) for the PK/PD targets in the plasma, %TC_f_ > MIC, and %TC_f_ > 4xMIC and, in the peritoneal compartment, %TC_f_ > MIC are presented in the Supplementary Materials.

## 3. Results

### 3.1. Clinical and Pharmacological Data

During the study period, 45 consecutive patients received an antibiotic therapy with PTZ while being treated by OA/VAC. Only one patient was excluded for non-conformity with the study protocol. The main characteristics of the population are summarized in Table 2. The observed pharmacokinetics of the PTZ administered continuously in critically ill patients with OA/VAC are depicted Table 1.

### 3.2. Pharmacokinetic Model Building and Monte Carlo Simulation

The piperacillin and tazobactam concentrations were described using a two-compartment model with linear elimination and proportional residual error. The sequential model covariate development is displayed in the Supplementary Materials.

Goodness-of-fit plots of the final pharmacokinetic models were considered acceptable, with good correlation coefficients between the observed concentrations and individual (respectively, R^2^ values of 0.967 and 0.811 for PIP in the plasma and peritoneal compartment and 0.991 and 0.800 for TAZ in the plasma and peritoneal compartment). Population and individual weighted residuals, normalized prediction distribution errors, and visual predictive checks are given in the Supplementary Materials. Most residuals were within the expected range (−2; +2).

The final PIP and TAZ population PK parameter estimates and their associated levels of precision are provided in Table 3. All were reliably estimated, with a percentage of standard error of <50%. More than 90% of the observed concentrations were contained within the prediction interval. Using the simulated concentration–time profiles at steady state, the calculated PTA for each PTZ dosing regimen (12 g/1.5 g/day, 16 g/2 g/day, and 20 g/2.5 g/day) in patients with various renal function (CRRT = group B; renal failure = group C; normal renal function and augmented renal clearance [ARC] = group A) are shown in Figure 3. The calculated fractional target attainment (FTA) for each PTZ regimen is given in the Supplementary Materials.

## 4. Discussion

Open abdomen and negative pressure therapy are surgical techniques used in critically ill patients with abdominal compartment syndrome (ACS) or other conditions that require temporary abdominal closure. These techniques can have an impact on the pharmacokinetics and pharmacodynamics of medications administered to these patients, especially distribution and elimination. To our knowledge, this is the first study aiming to characterize the PK/PD interactions induced by open abdomen and negative pressure therapy in critically ill patients treated by PTZ administered continuously and adapted to renal function.

This specific population was characterized by a wide inter-individual variability of pharmacokinetics parameters. In accordance with our results, creatinine clearance was the only covariate in describing piperacillin and tazobactam pharmacokinetic parameters and age end body weight were the two covariates in describing piperacillin pharmacokinetic parameters [4]. Compared with previous PK data, the estimated VD of PIP and TAZ in our population was not significantly higher than previously reported in critically ill patients, with estimations varying between 10 and 53 L for PIP and 25 and 31 L for TAZ according to El-Haffaf et al. [17]. Moreover, the intraperitoneal diffusion was evaluated at 88% for TAZ and PIP, in accordance with other studies [18,19,20]. Finally, our study suggested that the VAC-induced peritoneal clearance was negligible compared to renal clearance. Specific covariates such as the amount of fluids collected by the VAC did not improve the pharmacokinetic model building, suggesting that the influence of OA/VAC on PIP and TAZ PK parameters was not straightforward.

In this context, the desirable PK/PD target (i.e., peritoneal unbound concentration > 16 mg/L) may be achieved in most patients using a CL_CR_-pondered PTZ dosing regimen administered by continuous infusion (ranging from 12/1.5 to 20/2.5 g/day according to renal function). Our results thus emphasize the need for a higher-than-licensed dosing regimen in critically ill patients to obtain the desirable PK/PD target for tazobactam in the plasma and peritoneal compartment (i.e., plasma and peritoneal unbound concentration > 2 or 4 mg/L), especially for patients with normal renal function and augmented renal function.

Although predictive scoring systems can help the clinician to screen for ARC at the bedside where antimicrobial adjustments should be considered, measured CL_CR_ must remain the reference and should be monitored daily in at-risk patients [3,21]. The toxicity of piperacillin used in combination with tazobactam depends primarily on the plasma concentrations achieved; the duration of exposure; and individual patient characteristics, such as renal function. To assess the likelihood of reaching toxic concentrations, it is essential to know the toxicity thresholds associated with piperacillin. The following is an overview of known toxicity thresholds and important considerations for assessing the risk of toxicity at high doses. To study the probability of reaching toxic concentrations of piperacillin and, in particular, neurotoxicity, it is necessary to use toxicity thresholds based on Cmax or AUC as risk indicators, to adapt doses according to renal function and to consider prolonged administration schedules to avoid concentration peaks and, finally, to implement therapeutic drug monitoring, particularly in patients with risk factors such as renal insufficiency, to optimize the safety and efficacy of treatment [22,23].

Regardless of the dose used in piperacillin and the patient group (A, B, or C), the objective of PTA 100% fT > MIC is achieved both at the plasma level and at the peritoneal level for ECOFF at 8 mg/L (enterobacteria) from the dose of 12 g/1.5 g/day of piperacillin–tazobactam. On the other hand, for an ECOFF at 16 mg/L (Pseudomonas aeruginosa and Enterococcus faecalis) and to be as close as possible to the objective 100% fT > MIC, it is preferable to use a dose of 16 g/2 g/day or even 20 g/2.5 g/day of piperacillin–tazobactam. We have also demonstrated (unpublished data) that to achieve the PTA target of 85% fT > 4xCMI at the plasma level, the dose of 20 g/2.5 g/day of PTZ is essential for an ECOFF at 8 mg/L in group A of the patients. In this same group and at the dose of 20 g/2.5 g/day of PTZ, the PTA value is 50% fT > 4xCMI for an ECOFF at 16 mg/L.

Regardless of the dose used in piperacillin–tazobactam and the patient group (A, B or C), the objective of PTA 85% fT > MIC is achieved both at the plasma level and at the peritoneal level for an MIC of 2 mg/L from the dose of 12 g/1.5 g/day of piperacillin–tazobactam. On the other hand, to achieve this same PTA 85% fT > MIC objective for an MIC equal to 4 mg/L, the dose of 20 g/2.5 g/day of piperacillin–tazobactam is necessary.

Due to these complex pharmacokinetic changes and variabilities, individualized antibiotic dosing strategies are necessary in critically ill patients. Therapeutic drug monitoring (TDM) and the use of pharmacokinetic modeling and simulation techniques can help optimize antibiotic dosing regimens to achieve desired drug exposures in this population. It is crucial to involve a clinical pharmacist or infectious disease specialist experienced in managing critically ill patients to ensure appropriate antibiotic dosing and minimize the risk of treatment failure or toxicity.

RSE% is sometimes high for certain PK parameters. This high variability is due to the inclusion of peritoneal sampling in the models. This implies greater constraints on the compartments of the structural model and removes degrees of freedom from Bayesian estimators, even more in the context of continuous perfusion [24]. In addition, we have chosen to deal with free rather than total PTZ concentrations to account for variability due to plasma protein binding. Finally, these constraints bring a high degree of clinical relevance to the results and provide a clear answer to the hypotheses on the effect of OA/VAC.

However, the major limitation of our study is that we did not include patients with and without OA/NPT, precluding any comparison about the influence of OA/VAC on PTZ pharmacokinetics.

## 5. Conclusions

In conclusion, our study helps to characterize the PK/PD interactions in critically ill patients with open abdomen and negative pressure therapy and shows no significant changes in total distribution volume or extrarenal clearance. The OA/VAC system shows no impact on PTZ PK/PD. We can consider the dosing regimens for intra-abdominal infections based on empirical PK/PD target attainment at the peritoneal site. Our results emphasize the need for higher-than-licensed dosing regimens and therapeutic drug monitoring in critically ill patients at risk of underdosing despite a 20/2.5 g/day dosing regimen.

## Figures and Tables

**Table 1:** Non-compartmental pharmacokinetic data of PTZ in critically ill patients with OA/VAC.

	Piperacillin	Tazobactam
Observed concentration at steady state (mg/L)		
- Plasmatic concentration - Peritoneal fluid concentration	83 [52–100] 69 [43–61]	19 [12–25] 18 [7–24]
Drug underdosing	1 (2)	5 (11)
Peritoneal diffusion (%)	88 [73–98]	88 [68–104]
Calculated drug clearance		
- Renal clearance (mL/min) - Calculated VAC clearance (L/h)	129 [62–231] 0.6 [0.3–1.0]	65 [45–150] 0.7 [0.3–1.0]

Data expressed as median [25–75% interquartile] and number (percentage). Drug underdosing was defined by a peritoneal free concentration < 16 mg/L for piperacillin and 2 mg/L for tazobactam.

**Table 2:** Characteristics of the population.

	Overall Population *n* = 45
Demographic and anthropometric data at T_0_	
- Age (years) - Male sex - Body mass index (kg/m^2^) - Total body weight (kg) - Adjusted body weight (kg)	66 [57–72] 36 (80) 28 [25–30] 83 [71–92] 79 [69–87]
Medical history	
- SAPS 2 at ICU admission - Charlson comorbidity index	52 [39–71] 4 [3–5]
Initial surgical management and indication for OA/VAC	
- Emergency vs. elective surgery - Traumatic surgery vs. non-traumatic surgery - Prophylactic OA/VAC vs. secondary complication	33 (73) vs. 12 (27) 10 (22) vs. 35 (78) 37 (82) vs. 8 (18)
Type of intra-abdominal infection	
- Primary peritonitis - Secondary peritonitis - Acute mesenteric ischemia - Prosthetic vascular graft infection - Other	11 (24) 11 (24) 10 (22) 9 (20) 4 (9)
Microbiological documentation	
- Enterobacteriacae - Streptococcus/enterococcus species - Staphylococcus species - Anaerobic bacteria - Non-fermenting GNB - Absence of sterilization of peritoneal cultures at T1	34 (76) 17 (38) 11 (24) 8 (18) 1 (2) 18 (40)
Clinical and biological data at T1	
- Time between introduction of PTZ and T_1_ (hours) - Modified SOFA score - Serum albumin - Renal function ○ A: Normal renal function (60 < CL_CR_ ≤ 150 mL/min) ○ B: Need for CRRT ○ C: Moderate renal impairment (30 < CL_CR_ ≤ 60 mL/min) ○ D: Augmented renal clearance (CL_CR_ > 150 mL/min)	48 [48–72] 3 [3–7] 25 [20–28] 22 (49) 12 (27) 8 (18) 3 (7)
Patient’s outcome	
- Number of surgical revisions before fascial closure - Total duration of OA/VAC before fascial closure (days) - Intraperitoneal collection after abdominal closure - Secondary acquisition of resistance to PTZ - ICU length of stay - In-hospital mortality	2 [1–3] 4 [3–8] 11 (24) 5 (11) 15 [10–21] 8 (18)

Data expressed as median [25–75% interquartile] and number (percentage).

**Table 3:** Population pharmacokinetics parameters of unbound piperacillin and tazobactam.

	Piperacillin		Tazobactam	
Parameter	Value	SE (RSE %)	Value	SE (RSE %)
Fixed effects				
$V1_{pop}$ (L)	21.213	17.818 (84)	23.248	2.789 (12)
$\beta_{V1,\;age}$	−0.016	0.004 (29)	/	/
$\beta_{V1,\;weight}$	0.017	0.005 (30)	/	/
$V2_{pop}$ (L)	5	/	5	/
$Q_{pop}$ (L/h)	23.833	5.958 (25)	20	/
$Cl1_{pop}$ (L/h)	38.739	32,153 (83)	7.013	2.594 (37)
$\beta_{Cl1,\;Cl\;cat\;=\;A}$	0	0 (0)	0	0 (0)
$\beta_{Cl1,\;Cl\;cat=B}$	−2.016	0.443 (22)	−5.602	3.529 (63)
$\beta_{Cl1,\;Cl\;cat=C}$	−1.243	0.360 (29)	−2.303	0.667 (29)
$Cl2_{pop}$ (L/h)	5.567	0.612 (11)	3.003	0.360 (12)
Inter-individual variability				
ωV1	0.225	0.056 (25)	0.351	0.119 (34)
ωCl1	0.661	0.178 (27)	0.569	0.500 (88)
ωCl2	0.123	0.106 (86)	0.243	0.123 (51)
Intra-individual variability				
γCl1	0.391	0.160 (41)	0.967	0.348 (36)
Residual variability				
$b_{C1}$	0.207	0.028 (14)	0.371	0.063 (17)
$b_{C2}$	0.239	0.033 (14)	0.372	0.048 (13)

SE, standard error; RSE, relative standard error; V1, central volume of distribution; $\beta_{V1}$ impact of covariates on central compartment volume; V2, peritoneal volume of distribution; Q, intercompartmental clearance; Cl1, central compartment clearance; $\beta_{Cl1}$, impact of covariates on central compartment clearance (the value depends on renal category: normal renal function and augmented renal clearance [A], continuous renal replacement therapy [B], and moderate renal impairment [C]); Cl2, peritoneal compartment clearance; $b_{C1}$, parameter of the proportional residual error model on central compartment concentrations; $b_{C2}$, parameter of the proportional residual error model on peritoneal compartment concentrations.

## Data Availability

The datasets used and/or analysed during the current study are available from the corresponding author on reasonable request. The data will be made available on reasonable request.

## Supplementary Materials

The following supporting information can be downloaded at https://www.mdpi.com/article/10.3390/pharmaceutics16091191/s1, Figure S1: Observed concentration in the plasma (A,C) and peritoneal compartment (B,D) for piperacillin (upper panel) and Tazobactam (lower panel); Figure S2: Visual predic.ve check for PIP model in the (A) central compartment (Plasma) and (B) peritoneal compartment (Peritoneum); Figure S3: Observations vs. Predictions for PIP model in the (A) Central compartment (Plasma), Spearman: 0.991 and (B) peritoneal compartment (Peritoneum), Spearman: 0.8; Figure S4: Visual predic.ve check for TAZ model in the (A) central compartment (Plasma) and (B) peritoneal compartment (Peritoneum); Figure S5: Observations vs. Predictions for TAZ model in the (A) Central compartment (Plasma), Spearman: 0.967 and (B) peritoneal compartment (Peritoneum), Spearman: 0.811; Figure S6: Residuals Scatterplot (IWRES) for PIP model in the (A) central compartment (Plasma) and (B) peritoneal compartment (Peritoneum); Figure S7: Residuals Scatterplot (IWRES) for TAZ model in the (A) central compartment (Plasma) and (B) peritoneal compartment (Peritoneum); Table S1: Piperacillin and Tazobactam pharmacokinetic model compara.ve log-likelihood in (2*LL), A IC, and BIC.

## Abbreviations

ARC	augmented renal clearance
BMI	body mass index
CL	clearance
CL_CR_	creatinine clearance
CRRT	continuous renal replacement therapy
Css	concentration at steady state
ECOFF	epidemiological cut off
IAIs	intra-abdominal infections
ICU	Intensive Care Unit
MIC	minimum inhibitory concentration
NPDE	normalized prediction distribution error
OR	operative room
PD	Pharmacodynamics
PIP	Piperacillin
PK	pharmacokinetics
PTA	probability of therapeutic target attainment
PTZ	piperacillin–tazobactam
OA	open abdomen
SAPS 2	simplified acute physiology score 2
SOFA	sepsis-related organ failure assessment
TAZ	Tazobactam
TBW	total body weight
TDM	therapeutic drug monitoring
VAC	vacuum-assisted wound closure therapy
V1	central volume of distribution
VPC	visual predictive check
