# Supplementary material for: Pharmacokinetics of Piperacillin–Tazobactam in Critically Ill Patients with Open Abdomen and Vacuum-Assisted Wound Closure: Dosing Considerations Using Monte Carlo Simulation
Source: Pharmaceutics. 2024 Sep 9;16(9):1191. doi: 10.3390/pharmaceutics16091191 (PMC11434833; doi:10.3390/pharmaceutics16091191)
Supplement: Supplementary file 1 [file pharmaceutics-16-01191-s001.zip › pharmaceutics-3156592-supplementary.pdf]

additional material

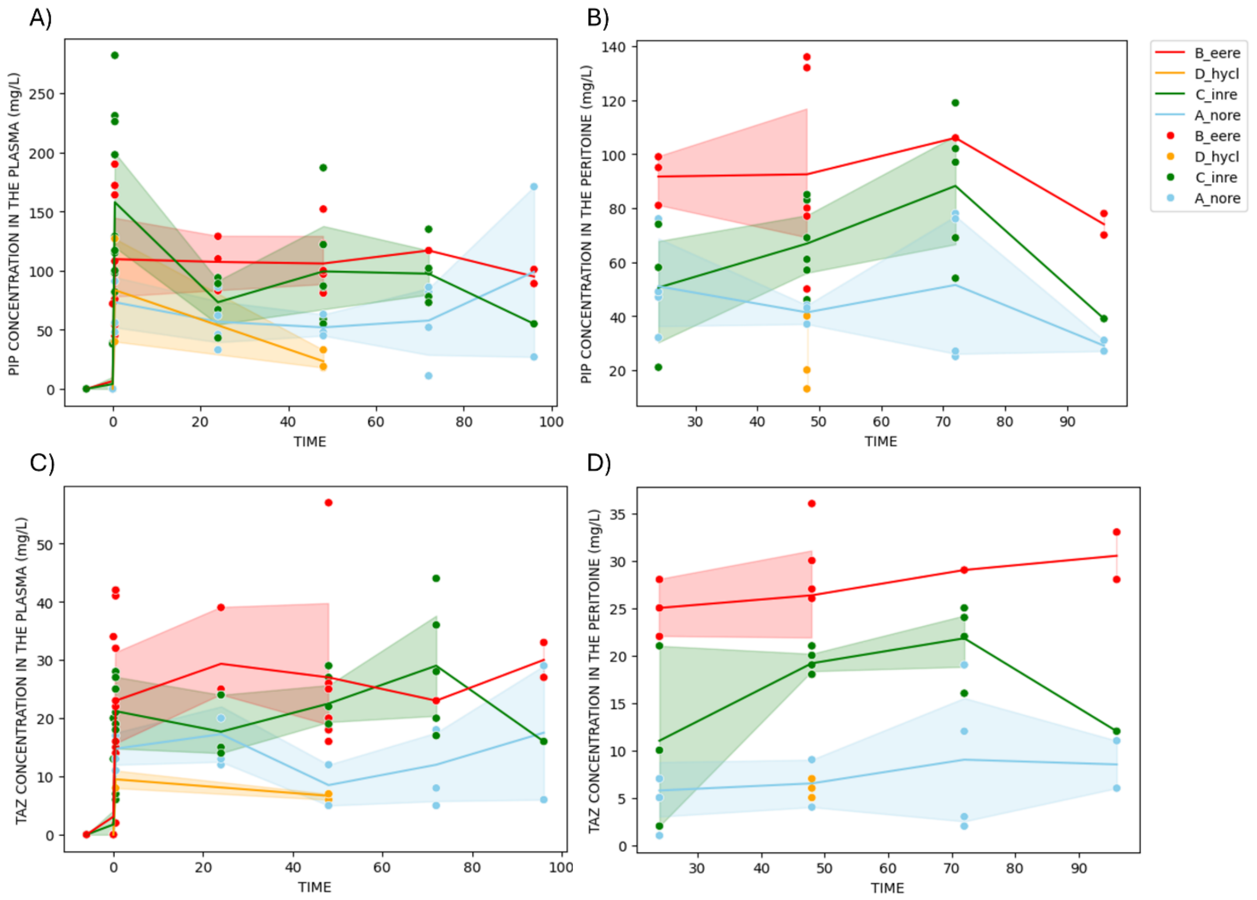

Figure S1. Observed concentration in the plasma (A,C) and peritoneal compartment (B,D) for piper acillin (upper panel) and Tazobactam (lower panel).

Table S1: Piperacillin and Tazobactam pharmacokinetic model comparative log-likelihood in (2*LL), A IC, and BIC.

Model description	2*LL	AIC	BIC
2 compartment model			
Covariate model with V2 fixed, age as a covariate on Cl1 and V1, weight on V1, categorical clearance on Cl1	-2027.7	-1997.7	-1960.6
Base model with random effect on all parameters; age as a covariate on Cl1 and V1, and weight on V1	-2007.3	-1977.3	-1940.1

Model description	2*LL	AIC	BIC
2 compartment model			
Covariate model with V2 and Q fixed, and categorical clearance on Cl1	-2223.3	-2201.3	2174.5
Base model with random effect on all parameters	-2215.1	-2195.1	-2170.7

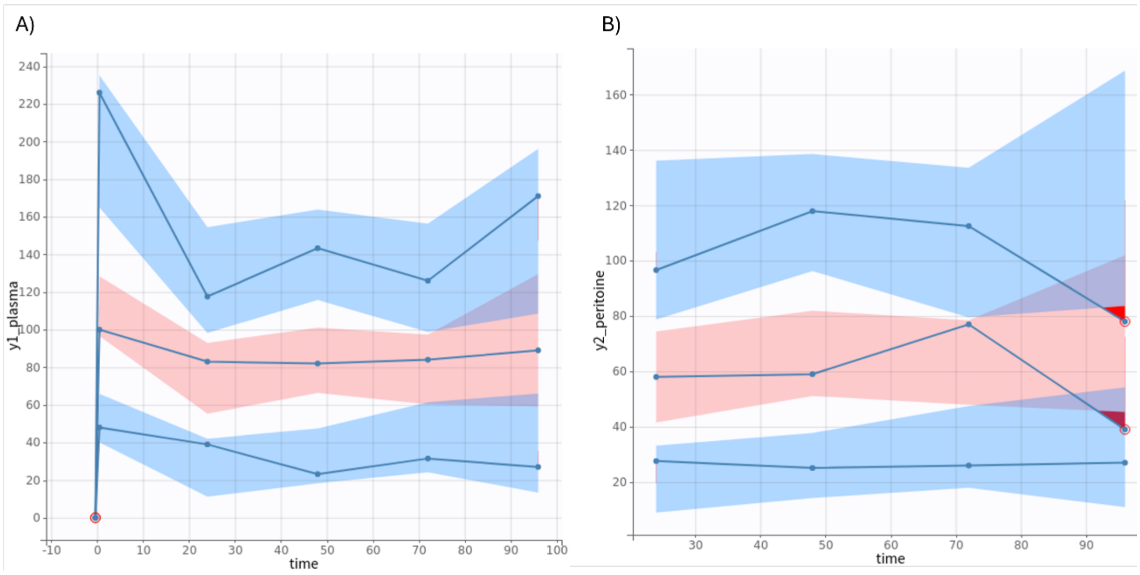

Figure S2. Visual predictive check for PIP model in the (A) central compartment (Plasma) and (B) peritoneal compartment (Peritoneum).

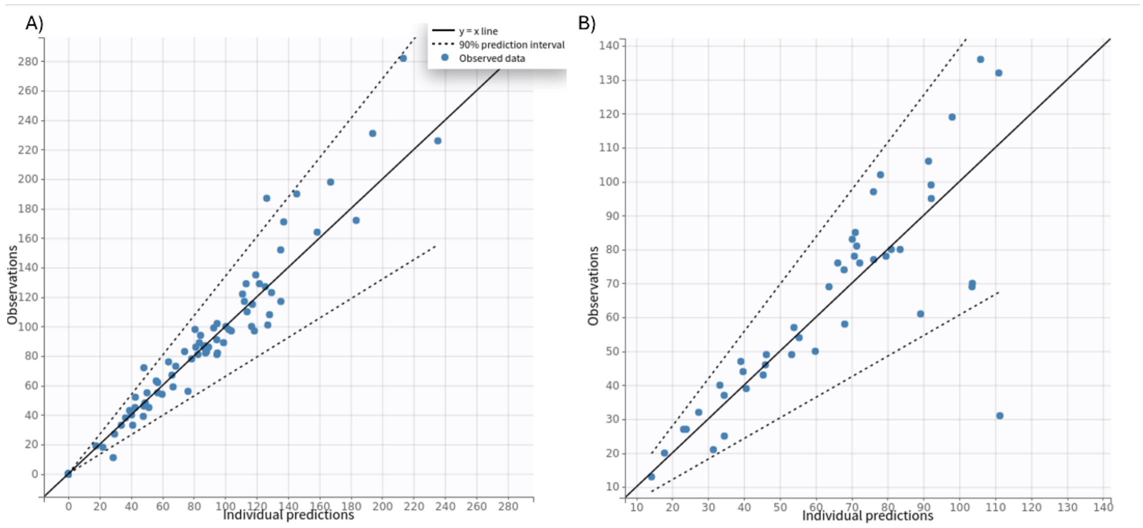

Figure S3. Observations vs Predictions for PIP model in the (A) Central compartment (Plasma), Spearman: 0.991 and (B) peritoneal compartment (Peritoneum), Spearman: 0.8.

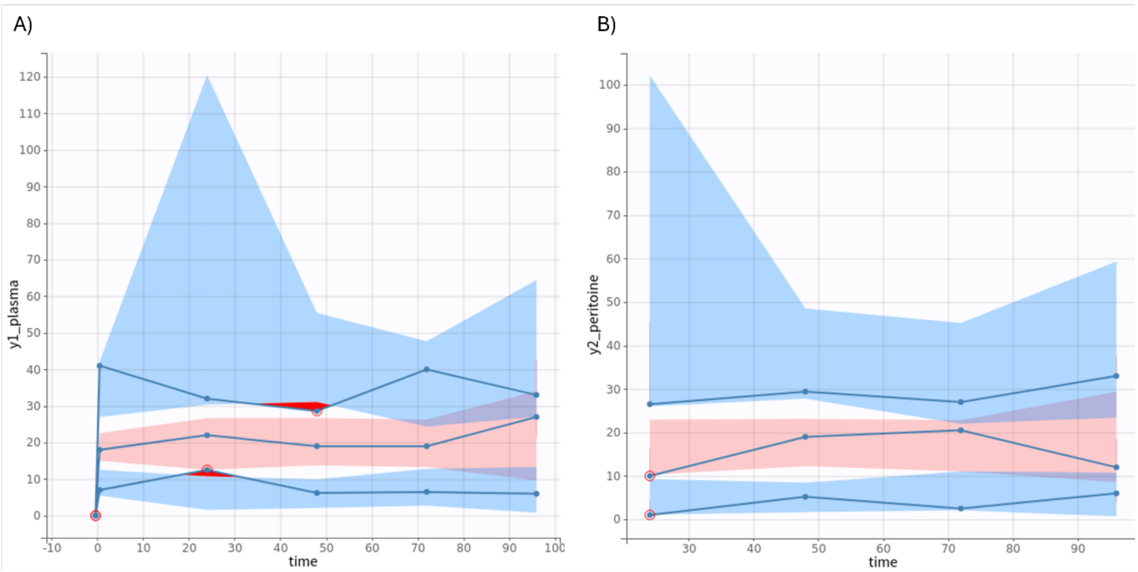

Figure S4. Visual predictive check for TAZ model in the (A) central compartment (Plasma) and (B) peritoneal compartment (Peritoneum).

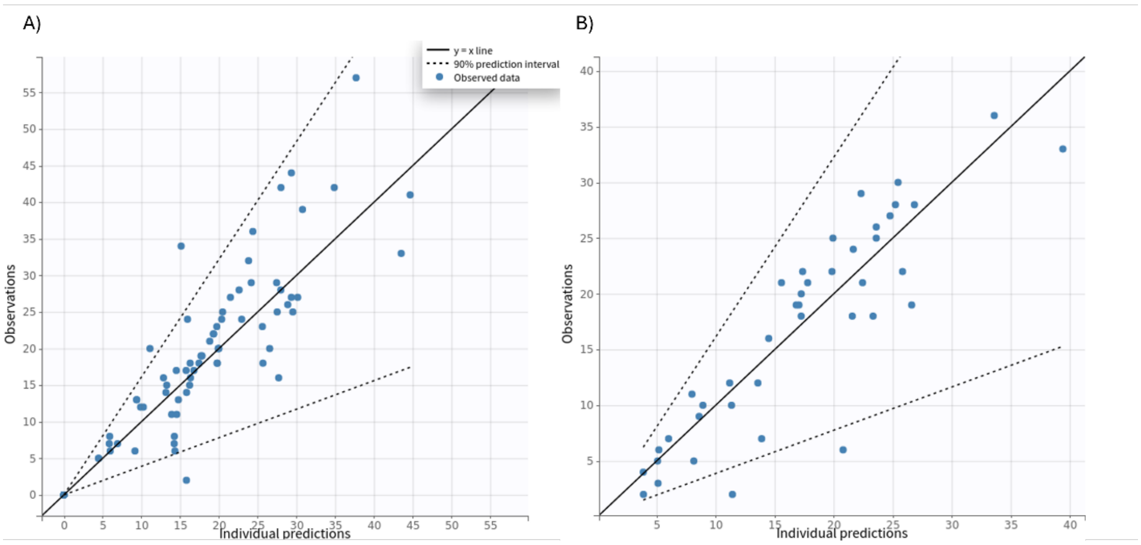

Figure S5. Observations vs Predictions for TAZ model in the (A) Central compartment (Plasma), Spearm an: 0.967 and (B) peritoneal compartment (Peritoneum), Spearman: 0.811.

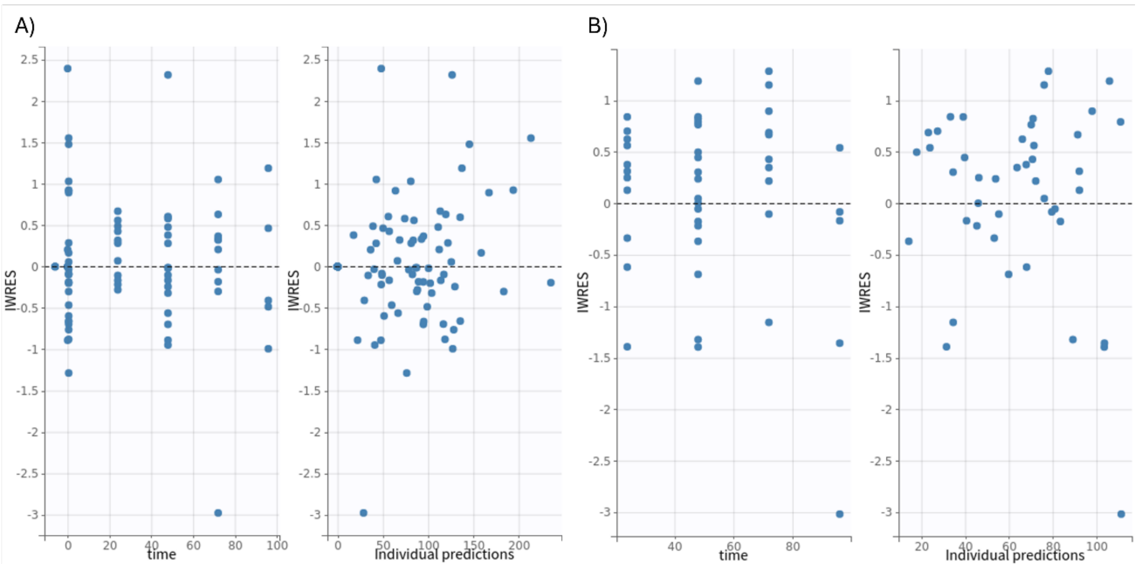

Figure S6. Residuals Scatterplot (IWRES) for PIP model in the (A) central compartment (Plasma) and (B) peritoneal compartment (Peritoneum).

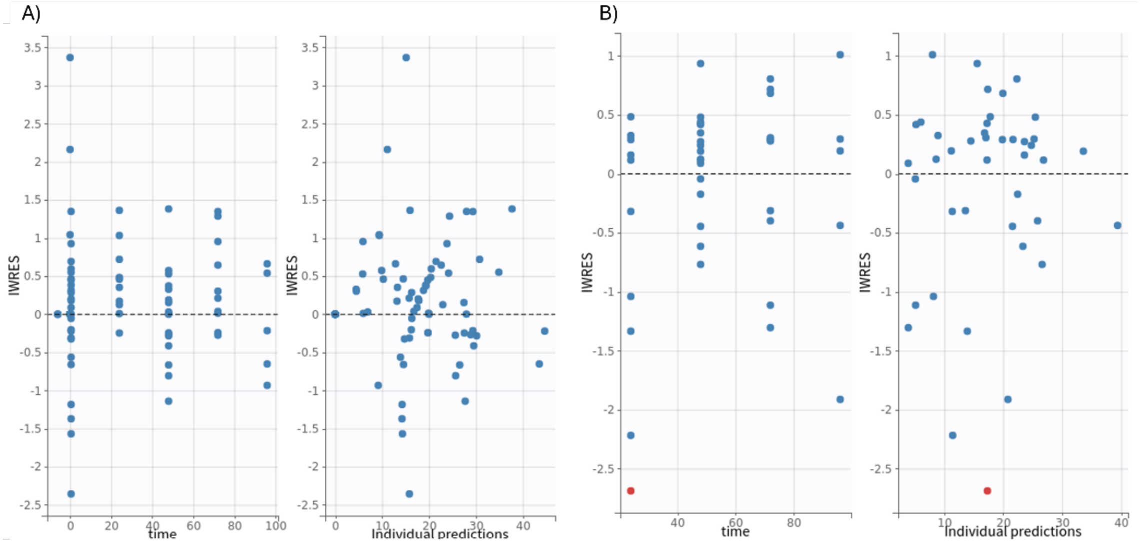

Figure S7. Residuals Scatterplot (IWRES) for TAZ model in the (A) central compartment (Plasma) and (B) peritoneal compartment (Peritoneum).
